# Expanding Our Knowledge of Menstrual Irregularities Reported by Females With Tuberous Sclerosis Complex

**DOI:** 10.3389/frph.2022.798983

**Published:** 2022-02-18

**Authors:** Kate Mowrey, Hope Northrup, Syed Shahrukh Hashmi, David Rodriguez-Buritica

**Affiliations:** ^1^Division of Medical Genetics, Department of Pediatrics, McGovern Medical School at the University of Texas Health Science Center at Houston (UTHealth Houston) and Children's Memorial Hermann Hospital, Houston, TX, United States; ^2^Pediatric Research Center, Department of Pediatrics, McGovern Medical School at the University of Texas Health Science Center at Houston (UTHealth Houston) and Children's Memorial Hermann Hospital, Houston, TX, United States

**Keywords:** menstrual irregularity, tuberous sclerosis complex, mTOR dysregulation, mTOR inhibitors, reproductive health

## Abstract

**Purpose:**

The purpose of our study is to expand the knowledge regarding intrinsic reproductive dysfunction in females with TSC and to explore the impact of mTOR inhibitors (mTORi) on menstrual irregularity in the Tuberous Sclerosis Complex (TSC) community.

**Methods:**

An electronic survey composed of author-designed questions set out to evaluate reproductive history, presence of menstrual irregularities, mTORi use, as well as maternal reproductive history among females with TSC.

**Results:**

Of the 68 responses from females with TSC regarding age of menarche, the average age was 12.3 years. 56.5% (*n* = 48) of respondents reported irregular menstrual cycles and noted a total of 102 menstrual irregularities. There was a cohort of 35 women with a reported history of mTORi use. Of these women, 68.6% (*n* = 24) reported irregular menstrual cycles after taking mTORi. In comparison, among the females with no history of mTORi use (*n* = 50) only 48% reported irregular menstrual cycles (*n* = 24).

**Conclusions:**

Our data expands the knowledge regarding intrinsic menstrual dysregulation present in women with TSC, demonstrates a rate of menstrual irregularities among females taking mTORi, and identifies a tendency toward early menarche that may be a previously unrecognized feature of TSC.

## Introduction

Tuberous sclerosis complex (TSC) is an autosomal dominant condition that is characterized by the growth of hamartomas across multiple organ systems, notably the skin, heart, brain, kidneys, and lungs. The estimated prevalence is 1 per 6,000 to 10,000 live births (1). Approximately two-thirds of cases are *de novo* and the remaining one-third are inherited (2, 3).

The molecular genetic etiology of TSC is pathogenic variants in the *TSC1* or *TSC2* gene, which encode for the proteins hamartin and tuberin, respectively. Hamartin and tuberin form heterodimers to regulate mammalian target of rapamycin complex 1 (mTORC1) (4–6). The mTOR pathway uses intracellular and extracellular signals to control cell growth, proliferation, and metabolism (7). The germline pathogenic variant in either *TSC1* or *TSC2* conveys a reduction in functional protein, therefore, the regulation of mTORC1 remains intact. The second somatic pathogenic variant in the remaining *TSC1* or *TSC2* allele leads to dysregulation of the mTOR pathway and contributes to the characteristic clinical stigmata associated with TSC (2, 8).

There has been minimal research thus far regarding reproductive manifestations in TSC, but it is known that intact mTOR activity is key in regulating several female reproductive processes including folliculogenesis, oocyte meiotic maturation, ovarian somatic cell proliferation, steroidogenesis, pubertal onset, ovarian aging, endometrium changes, and embryonic development (9). In particular, the use of conditional knockout models revealed the role of murine *Tsc1* and *Tsc2* genes in the ovaries. Knock-out mouse model for *Tsc1* and *Tsc2* were seen to have premature activation of the primordial follicles and loss of the follicular pool. Similar changes in humans would present with a phenotype characterized by premature ovarian insufficiency (POI) (10, 11). Evidence of this was supported by Gabitzsch et al. who performed a cross-sectional study, revealing women with a diagnosis of TSC self-reported significant menstrual irregularities (33%), elevated rate of miscarriage (41%), and histories suggestive of several reproductive disorders (12). Another layer of complexity is the common use of mTOR inhibitors (mTORi) to reduce the size of TSC-related hamartomas across all organ systems. A study of individuals with Autosomal Dominant Polycystic Kidney Disease (ADPKD) noted that after 19 months on an mTORi, approximately 52% of their female patients reported menstrual cycle abnormalities. The percent affected was significantly increased compared to their control group, whom 17% reported menstrual cycle abnormalities (13). Sparagana et al. pooled data from two mTORi (everolimus) clinical trials (EXIST 1 and 2) and reported that 38.4% of female TSC participants experienced at least one episode of menstrual irregularity while taking an mTORi compared to 2.3% of the females in the placebo group (14). Lastly, it is important to note that there is no data from previous studies that reveal insight on if the use of mTORi impacts the timing of menarche.

Another consideration is the suspected teratogenic effect of mTORi during pregnancy. Currently, there are limited reports about the use of mTORi during human pregnancy, and animal studies indicate adverse effects in rats, therefore, pregnancy is currently discouraged in individuals under therapy (15). As a result, many females with TSC are concurrently taking oral contraceptive pills (OCPs) and the influence of the combination of both therapies has yet to be investigated at this time.

Our study seeks to expand the knowledge regarding intrinsic reproductive dysfunction in females with TSC and to explore the impact of mTORi on menstrual irregularity in the TSC community.

## Materials and Methods

### Study Design and Population

This study was cross-sectional and used a web-based approach to survey females with TSC aged 8 years and older. The study was approved by the Institutional Review Board of University of Texas Health Science Center at Houston (HSC-MS-19-0273). The one-time 10-min electronic questionnaire was designed using Qualtrics online software (Qualtrics, Provo, UT). The eligibility criteria for participation were to be a female with a molecularly or clinically confirmed diagnosis of TSC and at least 8 years old. Individuals who were 8–17 years old or had cognitive impairment at any age required that the questionnaire be filled out by their parent or caregiver. Data collection occurred from June 2019 to December 2019. Electronic consent was collected prior to starting the survey. The data was collected from two primary sources: email and/or social media posts by the Tuberous Sclerosis (TS) Alliance (now named the TSC Alliance) and in-person at a single TSC clinic. The TS Alliance sent the survey out through their listserv as well as their social media platforms providing a hyperlink to our survey a minimum of three times. The latter set of participants were seen in a single multidisciplinary clinic at McGovern Medical School at the University of Texas Health Science Center at Houston. Females that met the eligibility criteria were offered the chance to participate in the study. If they agreed to participate, they were given an electronic device to self-administer the survey. Survey respondents were given the opportunity to be compensated for their time by providing a valid mailing address to receive a $5.00 gift card. The data collected by the survey was kept confidential, de-identified, and password protected. We received 109 surveys, but nine were excluded due to lack of information or not meeting eligibility criteria, providing a final sample size of 100.

### Questionnaire Design

The questionnaire was designed by the authors after a thorough literature search regarding menstrual irregularity in TSC as well as menstrual irregularity in conjunction with the use of mTORi. The structure of the survey included five main parts: demographics, reproductive history, history of menstrual irregularities, history of mTORi use, and maternal reproductive history. The demographic section collected information regarding diagnosis of TSC, sex, current age, diagnosis of intellectual disability, residence, ethnicity, and education. The reproductive history section obtained information about use of OCPs, history of pregnancy, history of infertility, presence or absence of menarche and menopause, age of menarche, and menopause, as well as whether the menstrual cycles were regular or irregular. If irregular, respondents were given seven options to characterize their type of menstrual irregularity and were allowed to select more than one type of menstrual irregularity. These seven options were condensed into five categories: short menstrual cycles, infrequent menstrual cycles, secondary amenorrhea, delayed puberty, and heavy menstrual bleeding. The mTORi section asked each respondent about previous use of an mTORi. If the respondent had taken an mTORi, they were asked about if their menstrual cycle was regular or irregular before and after using an mTORi. If irregular, they were given the same seven previously stated options. Lastly, maternal reproductive history gathered information regarding a maternal diagnosis of TSC, age of menarche, regular or irregular menstrual cycles, presence or absence of menopause, and age of menopause, if applicable. If irregular menstrual cycles were selected, the respondents were given the same seven previously stated options.

### Menstrual Irregularities

The respondents were first asked if their menstrual cycle was regular or irregular 2 years after menarche, as anovulatory and irregular cycles are expected during this time window (16). Regular menstrual cycles were defined as occurring every 21–35 days. Irregular menstrual cycles were defined as occurring less than every 21 days or more than every 35 days. The following five menstrual irregularity categories were each individually defined within the survey. Short menstrual cycles were defined as menstrual cycles that are less than 21 days apart. Secondary amenorrhea was defined as regularly missing at least three menstrual cycles in a row. Infrequent menstrual cycles were defined as regularly going more than 35 days without a period. Delayed puberty was defined as any female who had not had menarche by 15 years of age. Heavy bleeding was defined as menstrual bleeding that lasts more than 7 days, changing tampons and/or pads after less than 2 h, or passing blood clots the size of a quarter or larger (17).

### Statistical Analyses

Data was assessed and described as frequencies (with percentages) if categorical and as means (with standard deviations, sd) or medians (with interquartile ranges, IQR) if continuous in nature. The total range of the values for the continuous variables was also described. The presence of menstrual irregularities were reported as frequencies (with percentages). Comparison of menstrual irregularities before and after mTOR use was performed using McNemar's test. Paired *t*-tests were utilized to compare the ages of menarche and menopause of the patients (daughters) to those of their mothers. All analyses were performed in Stata v.14 (Stata, College Station, TX). Statistical significance for comparative analysis was assumed at a Type I error rate of 5%.

## Results

### Demographics

Our final sample size was 100. The median age of our cohort was 27.5 years with an age range from 9 to 58 years and interquartile range of 16–37 years. The majority of respondents were Caucasian and from the United States. Education was observed to vary and correlate appropriately with the age of the respondent. Forty-one percent of respondents had intellectual disability. Of the 100 respondents, 43% were answered by parents and/or caregivers while the remaining 57% of respondents answered their own questions. The remaining demographic information is presented in Table 1.

**Table 1:** Demographics of all respondents, *n* = 100.

		Frequency^*^
Age (years), median (IQR)^†^		27.5 (16–37)
Race, *n*		
	Asian	2
	African American	7
	Caucasian	80
	Other	6
	Multiracial	5
Country of origin, *n*	
	United States	87
	Canada	4
	Mexico	1
	Other	8
Highest level of education, *n* (%)^**^	
	High school or less	24 (33)
	Trade school or some college	17 (23)
	Bachelor's degree	13 (18)
	Graduate/Professional degree	19 (26)
Presence of intellectual disability, *n*		41
Previous use of mTORi inhibitor, *n*		45
Age of initiation of mTORi use, median (IQR)^†^		25 (13–35)

* *Data reported as frequency unless otherwise specified*.

** *Data shown for respondents aged 17 or older, n = 73*.

† *IQR = interquartile range*.

### Menarche and Menopause History

There were 68 responses from individuals with a diagnosis of TSC and their unaffected mothers with information regarding their age of menarche. The mean age of menarche for females with TSC was 12.3 years (sd: 2.0) with a range of 9–16 years. There were 14 females with TSC that experienced menopause, 8 (57%) of whom had a history of taking an mTORi. For these 14 individuals, the median age of menopause was 51 years with a range of 39–55 years and an interquartile range of 50–52.

### Comparison Between Respondent Data and Maternal Data

Table 2 summarizes the average age of menarche (*n* = 68) and menopause (*n* = 12) in the respondents compared to their unaffected mothers, median age of menopause in maternal data (*n* = 72), as well as the average difference in menarche age between respondents and their unaffected mothers.

**Table 2:** Comparison between respondents and maternal data.

	**Individual with TSC**	**Unaffected mothers**	***p*-values**
Average age of menarche^*^	12.3 years (sd 2.0)	13.0 years (sd 1.7)	0.005
Average difference in menarche age^†^	−0.8 years (CI: −1.3 to −0.2)		
Average age of menopause^**^	51 years (IQR 50–52)	47.5 years (IQR 30–55)	0.097
Median age of menopause^††^		50 years (IQR 46–52)	

* *n = 68*.

** *n = 12*.

† *Difference in Age = patient's age at menarche – maternal age at menarche*.

†† *n = 72*.

When limited to patients who had no history of mTORi use before menarche (*n* = 61), the average difference-in-age was −0.6 years (95% CI: −1.2 to −0.1) (paired *t*-test *p* = 0.024). There were only seven patients who reported mTORi use prior to menarche, and although the magnitude of the difference-in-age at menarche was larger at −1.9 years (95% CI: −3.8 to 0.1) it failed to reach statistical significance (paired test *p* = 0.059). A comparison of the difference-in-age yielded no statistically significant difference between patients who used mTORi prior to menarche and those that didn't (*p* = 0.147).

### Menstrual Irregularities

Assessment of baseline regularity of menstrual cycle was performed on 85 individuals. These included 50 respondents that had no history of mTORi use. Additionally, included was the menstrual history prior to and after starting an mTORi for 35 patients. Overall, there were 45 individuals in our cohort that reported use of an mTORi with a median age at mTORi initiation of 25 years and a range of less than 1 year to 53 years of age (IQR: 13–35). However, 10 of them were excluded from the analysis on baseline menstrual irregularities either due to lack of menarche, being within 2 years of menarche, or inaccurate/incomplete data prior to mTORi use.

Slightly more than half of the 85 respondents (*n* = 48, 56.5%), reported irregular menstrual cycles (Table 3). There was no statistically significant difference in the frequency of each specific menstrual irregularities between the patients who had no history of mTORi and those present after initiating mTORi therapy (*p* > 0.05 for all).

**Table 3:** Frequency of menstrual irregularities in respondents with and without a history of mTORi use.

	**No history of mTOR**	**History of mTOR**	**All**
Irregular	48% (*n* = 24)	68.6% (*n* = 24)	56.5% (*n* = 48)
Regular	52% (*n* = 26)	31.4% (*n* = 11)	43.5% (*n* = 37)
Total *n*	*n* = 50	*n* = 35	*n* = 85

However, there was a statistically significant difference in the prevalence of menstrual irregularities between the respondents who had no history of mTORi (*n* = 24, 48%) and the respondents with a history of mTORi (*n* = 24, 68.6%) (*p* = 0.017).

The concordance of cycle status before and after mTORi use was also evaluated. Of the 18 respondents (out of 35) that had regular cycles before mTORi use is located in Table 4, with a trend that was statistically significant (McNemar test *p* = 0.013) resulting in a higher prevalence of irregular menses after initiating mTORi.

**Table 4:** Comparison between regular and irregular cycles before and after mTORi use.

**Menstrual cycle**	**Before mTORi**	**After mTORi**	Transformation^*^
Regular	18	8	2 respondents became regular
Irregular	17	26	11 respondents became irregular
Total (*n*)	35	34^**^	13

* *Transformation column indicates the number of respondents that indicated a change in regularity after initiating mTORi therapy*.

** *One individual responded before mTORi use, but not provide data for after mTORi*.

**Figure 1:**
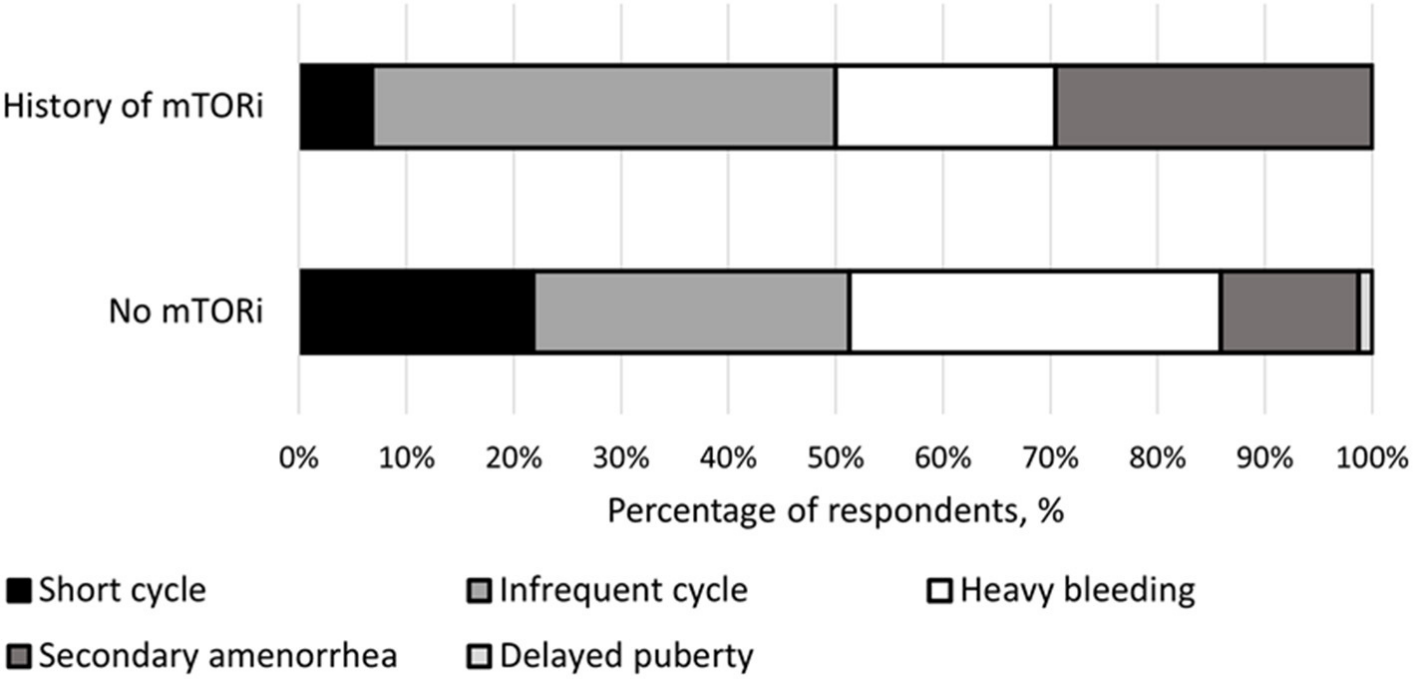
Breakdown of menstrual irregularities between females with a history of mTOR inhibitor (mTORi) use and without a history of mTORi use. Furthermore, the females that had a history of mTORi use had their menstrual irregularities further broken down to those present before and after starting mTORi therapy.

The type of menstrual irregularities reported by the 39 respondents after mTORi use were as follows: nearly half (*n* = 19, 49%) had infrequent menses, a third (*n* = 13, 39%) had secondary amenorrhea, a quarter (*n* = 9, 23%) had heavy bleeding, and 8% (*n* = 3) reported short menstrual cycles. For females not receiving mTORi, the following menstrual irregularities were noted: 24.1% (*n* = 14) with infrequent menses, 6.9% (*n* = 4) with secondary amenorrhea, 46.6% (*n* = 27) experienced heavy bleeding, 20.7% (*n* = 12) had short menstrual cycles, and one with delay puberty (1.7%) (Figure 1).

There were five females that reported starting an mTORi prior to menarche. Of these, 40% (*n* = 2) have regular menstrual cycles and the remaining 60% (*n* = 3) currently have irregular cycles but are still within their first 2 years since menarche.

### Oral Contraceptives and Reproductive History

**Figure 2:**
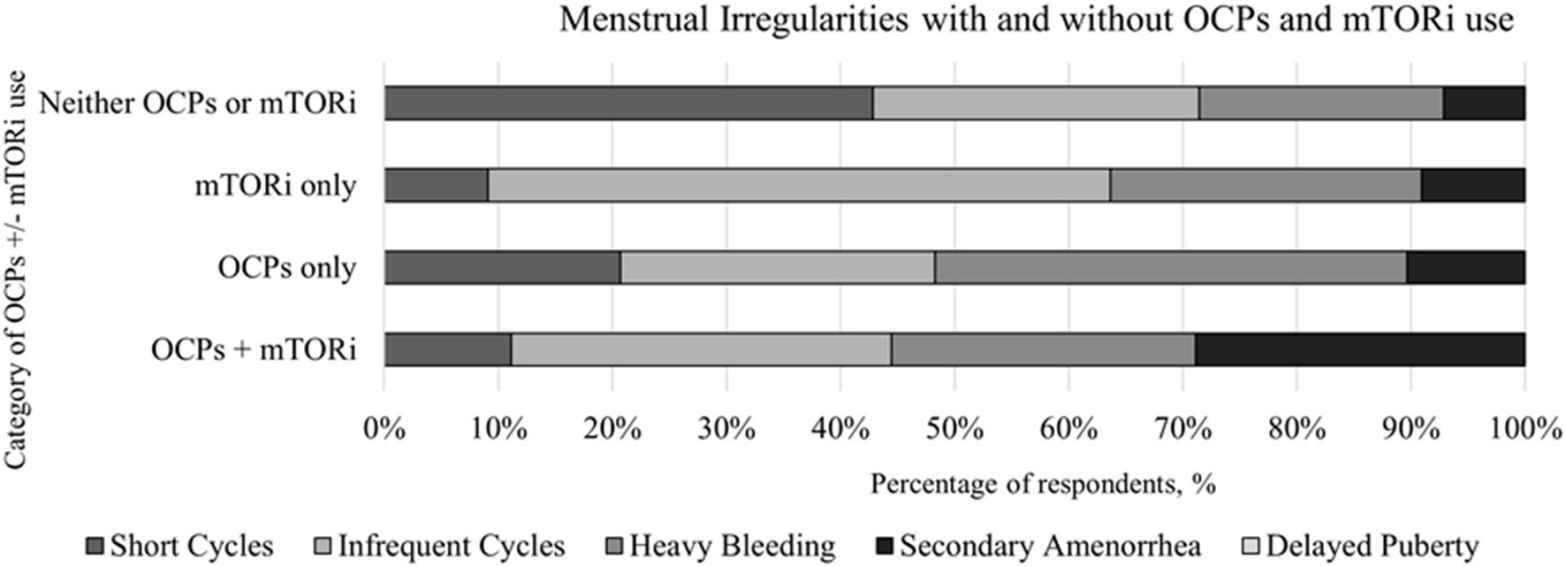
Comparison of the menstrual irregularities present in individuals with and without the use of oral contraceptive pills (OCPs) and mTOR inhibitors (mTORi) with further subcategorization of before and after the initiation of mTORi.

We obtained 98 responses regarding use of OCPs. Slightly less than two-thirds (*n* = 66) reported previous use of OCPs while the remaining 36% (*n* = 32) never used OCPs. Of the individuals with a history of using OCPs (n = 66), 45% (*n* = 30) had a history of using a mTORi. Similar proportion of mTORi use were seen in the 32 respondents with no history of OCPs, with 44% (*n* = 14) reporting a history of using a mTORi. The specific menstrual irregularity observed are depicted in Figure 2.

## Discussion

This study expands on the foundation of knowledge regarding menstrual irregularities in women with TSC by using self-reported data and comparing it to maternal data. To further describe potential factors influencing menstrual irregularities among women with TSC, we analyzed information on the use of mTORi as well as OCPs that could influence the rate of menstrual irregularities beyond the intrinsic nature of the disease.

Extrapolation of cellular and animal data to the human phenotype predicts some of the reproductive abnormalities observed in *TSC1* and *TSC2* heterozygous females (12). Even though the mTOR pathway is a key regulator of the onset of puberty, there is no evidence of early reproductive abnormalities such as abnormal onset or timing of puberty among females with TSC (18). The most likely explanation for this phenomenon is the paucity of studies on the topic. Interestingly, in our cohort, the average age of menarche was 12.3 years (sd 1.7) and is also not statistically significant from the general population and similar to that of previously studies (19–21). However, when compared to their mothers, the average age of menarche in females affected with TSC (12.3 years) occurs slightly earlier than the average age of menarche of unaffected mothers and is statistically significant (13.0; sd:2.0 and *p*-values: 0.005). This is the first instance that a trend toward earlier menarche has been reported in association with TSC. Since heritability of age of menarche has been estimated to be around 0.57 (22), the age of menarche of a woman is expected to be delayed by 3.4 months for every year increase in the mother's menarche. Interestingly, the trend identified in our study is in the opposite direction. The age of menarche on individuals treated with mTORi was reported on seven individuals and although the magnitude of the difference-in-age of menarche was larger, it failed to reach statistical significance (*paired test p* = *0.059*). The exact reasoning behind the significant difference in menarche among the group that received mTORi remains unclear and may reflect females with TSC requiring mTORi treatment may represent individuals with a higher burden of the disease and, therefore, higher effects of mTOR dysregulation. Even though this finding needs additional confirmation, intra-familial differences in the age of menarche with a tendency toward earlier menarche in affected females could be explained by intrinsic dysregulated mTOR pathway resulting in the acceleration of follicular activation and modify by use of mTORi. Overall, this is a subtle and novel observation as previous studies did not compare the age of menarche of females with TSC to the age of menarche of their unaffected mothers.

After puberty, it is clinically evident that women with TSC intrinsically present with manifestations of abnormal ovarian function given the previous documentation of increased rates of menstrual irregularities, miscarriages, and infertility (12, 14). This is further supported here in this study by the rate of menstrual irregularities in the non-mTORi group. To characterize these abnormalities further, our study set out to differentiate between the types of menstrual irregularities intrinsically associated with TSC and those associated with the use of mTORi. We confirmed the previously described higher rate of menstrual irregularities in subjects with TSC compared to the general population. The rate of menstrual irregularities for the groups representing the intrinsic effects of TSC, including (1) respondents with no history of mTORi and (2) respondents with history of mTORi use—prior to initiating therapy, was 48% (*n* = 24) and 49% (*n* = 17), respectively. Both rates are higher than the general population rate of 5–33.5% (23) and 33% for TSC subjects indicated in previous studies (12, 14). Furthermore, we identify that mTORi use plays an important role in the genesis of menstrual irregularities as its rate increases for subjects treated with mTORi in comparison to the non-treated group (48 vs. 74%; *p* = 0.001). We also documented a change in the pattern of menstrual irregularities after the use of mTORi. With frequent cycles and secondary amenorrhea being more common after initiation of treatment. The most common intrinsic menstrual irregularity was heavy bleeding. The higher rate of menstrual irregularities captured in our study may be attributable to participants allowed to pick multiple types of menstrual irregularities. Another factor that might explain the differences between the studies is the average age of the cohorts as the menstrual cycle patterns and the rate of menstrual irregularities change with age. The median age was 27.5 years in our study, while the average age was 42 years in Gabitzsch et al. (12) and median age was 23 years in Sparagana et al. (14).

The previous report by Gabitzsch et al. (12) indicated that women with TSC present with early menopause and POI (24). In our cohort, 12 respondents had experienced menopause with the average age of menopause reported as 51 years (39–55 years, *p*-value = 0.096). Of this subset of respondents, the average age of maternal menopause was 47.5 years (30–55 years). Even though we found concordance between the ages of menopause in our respondents compared to their mothers, these results contrast with the earlier age of menopause reported by Gabitzsch et al. (12). Our results as well as Gabitzsch et al. (12) (*n* = 4) are derived from a small number of respondents that leads to limited ability to draw strong conclusions from both studies. However, 45% (*n* = 45) of our cohort reported previous use of mTORi and 66% (*n* = 66) reported previous use of OCPs. The use of mTORi may have played a role in the later age of menopause observed in our study; however, OCPs do not seem to affect the age of menopause based on previous report of a large cohort by Langton et al. (25). This difference between our findings and previous reports needs additional investigation with a larger cohort size.

In summary, we confirmed and expanded on the intrinsic menstrual dysregulation that are present in women with TSC and identified a tendency toward early menarche that may be a previously unrecognized feature of TSC. Our data was collected via an online questionnaire, and we included data on unaffected mothers that allowed for intra-familial comparisons; however, recall bias of menarche and menopause age could be a source of error in our study. Notably, it is at those ages, during the early puberty and the end of the reproductive age, where we found most of the differences from previous reports. Additionally, we did not inquire about the presence or absence of seizure medications, presence or absence of uterine fibroids or surgical procedures such as hysterectomy or oophorectomy as these could be a factor on menstrual irregularity and menopause, respectively. Lastly, our conclusions are limited for those ages as the number of subjects in those age intervals were smaller than the rest of the cohort.

Future studies need to include larger groups of individuals with TSC to address some of the unresolved aspects in these emerging phenotypes. It is our opinion that there needs to be a delineation in the abnormalities seen during early puberty and menopause, as none of the studies thus far had a large enough number of subjects in the studied age groups to come to strong conclusions. This approach could pave the way for treatment options to improve the reproductive health of women with TSC or any other condition in which there is a dysregulation of the intracellular mTOR pathway.

## Data Availability Statement

The raw data supporting the conclusions of this article will be made available by the authors, without undue reservation.

## Ethics Statement

The studies involving human participants were reviewed and approved by Institutional Review Board of University of Texas Health Science Center at Houston (HSC-MS-19-0273). The patients/participants provided their written informed consent to participate in this study.

## Author Contributions

KM and DR-B: conceptualization and study design, manuscript writing, data collection, statistical analysis, and manuscript editing. HN: conceptualization and study design, data review, and manuscript editing. SH: statistical analysis and manuscript editing. All authors contributed to the article and approved the submitted version.

## Funding

The funding support from the UT Tuberous Sclerosis Complex Center Endowment Fund (#0007183).

## Conflict of Interest

The authors declare that the research was conducted in the absence of any commercial or financial relationships that could be construed as a potential conflict of interest.

## Publisher's Note

All claims expressed in this article are solely those of the authors and do not necessarily represent those of their affiliated organizations, or those of the publisher, the editors and the reviewers. Any product that may be evaluated in this article, or claim that may be made by its manufacturer, is not guaranteed or endorsed by the publisher.
